# Endomyocardial fibrosis in Sudan: clinical and echocardiographic features

**DOI:** 10.5830/CVJA-2016-079

**Published:** 2017

**Authors:** Siddiq Ibrahim Khalil, Suha Khalil, Salma El Tigani, Hanan A Saad

**Affiliations:** Department of Medicine, University of Medical Sciences and Technology, Khartoum, Sudan; The Heart Clinic, Khartoum, Sudan; Amal National Hospital, Khartoum, Sudan; Academy Teaching Hospital, Khartoum, Sudan

**Keywords:** endomyocardial fibrosis in Sudan, apical fibrosis ventricular cavity obliteration, endocardial fibrous shelf, endomyocardiopericarial fibrosis

## Abstract

**Objective:**

Endomyocardial fibrosis (EMF) is a rare disease and is often an underdiagnosed and forgotten cardiomyopathy. The objective of this study was to document the current frequency of EMF in Sudan by defining and selecting cases from patients attending the echocardiography laboratory. Additionally we aimed to create an EMF registry for Sudan.

**Methods:**

The study started in January 2007 and is on-going. All the patients attending our echocardiography clinics in four different hospitals in Khartoum, Sudan, were included. Transthoracic echocardiography was used as the main diagnostic and selection tool. The diagnosis of EMF was based on predefined criteria and definitions, and was further supported by additional clinical, ECG, laboratory and chest X-ray findings.

**Results:**

Out of 4 332 cases studied, 23 (0.5%) were found to have features of EMF. Females constituted 52% and the age range was 24 to 67 years. All patients presented with dyspnoea grades III–IV. Advanced heart failure with gross fluid overload was seen in 54% of cases and ascites was seen in 30%. EMF was biventricular in 53%, left ventricular in 29% and right ventricular in 18% of cases. Apical and ventricular wall fibrosis was found in all cases, followed by atrial enlargement, atrioventricular valve incompetence, ventricular cavity obliteration, restrictive flow pattern and pericardial effusion. Additional echocardiographic features are defined and discussed.

**Conclusion:**

Although a rare disease, cases of EMF can be identified in Sudan if a high index of suspicion is observed. New echocardiographic features of ventricular wall layering, endocardial fibrous shelf and endomyocardiopericarial fibrosis were identified and are discussed.

## Objective

Endomyocardial fibrosis (EMF) is a form of cardiomyopathy characterised by fibrosis and thickening that distinctly involves the ventricular apex and walls. It is one of the common causes of restrictive cardiomyopathy and is frequently underdiagnosed and occasionally mislabelled as rheumatic valvular disease or hypertrophic cardiomyopathy.

EMF was described by Loffler in 1936 in a patient with associated eosinophilia,^1^ and in Africa in 1946 by Bedford and Konstam,^2^ but its clinicopathological features were first recognised by Davies in Uganda in 1948.^3^ Although sporadic cases with similar clinical and pathological features have since been reported from other parts of the world, the majority of reported cases have come from West and Central Africa. In Uganda, it accounted for 25% of cases reported for echocardiography, and for 20% in a random population sample in Mozambique.^4^,^5^ EMF has also been reported from other subtropical countries such as Egypt^6^ and Nigeria, and some sub-Saharan countries,^7^,^8^ Brazil, and Kerala in India,^9^,^10^ It is exceedingly rare in Europe and NorthAmerica; however a few cases have been reported in China^11^ and Japan.^12^

The aetiology of EMF is unknown, however on an epidemiological basis, it behaves like a vector-transmitted disease. In addition to geography, several factors have been associated with the pathogenesis of EMF in Africa, including ethnicity, poverty, diet, age and gender, infection and eosinophilia.^3^ There is now evidence that the initial heart lesion in EMF may be associated with abnormalities of the eosinophils, although eosinophilia is common in many tropical regions where EMF does not appear to be prevalent. This suggests that other factors, possibly immunological in nature, are also necessary to determine the prevalence of the disease in a particular location.^3^,^6^

There is agreement among researchers that African endomyocardial fibrosis is a distinct entity and, despite the similarity in pathological features with Loffler’s endocarditis and the cardiac lesions seen in eosinophilic leukaemia or reactive eosinophilia, there is no hard evidence to suggest that African endomyocardial fibrosis is a variant of Loffler’s disease.^13^-^15^

Despite the lack of evidence to pinpoint the aetiology of EMF, some progress has been made in the diagnosis as well as in medical and surgical treatment. In earlier reports, the diagnosis of EMF was based on post mortem findings and clinical correlations but presently echocardiography is the standard diagnostic tool. Recently, cardiac magnetic resonance imaging (CMR) emerged as an additional tool to define the primary and secondary structural and functional abnormalities of EMF. CMR with gadolinium enhancement seems ideally suited to diagnose this condition and monitor response to medical and/or surgical therapy.^16^

Medical treatment of EMF entails reduction of fluid overload with diuretics and preload with nitrates. Rate and rhythm control may help in patients presenting with tachyarrhythmias, including atrial fibrillation. Surgical treatment of EMF was practiced for many years and consisted of removal of fibrosis from both ventricles, and atrioventricular valve replacement. Mitral or tricuspid annuloplasty has had only limited success.^17^

In Sudan, EMF was first described by O’Brien in 1954 and later by El Hassan, who had carried out post mortem studies in 133 cases of cardiovascular deaths. Among these, six out of 13 cases of cardiomyopathy had EMF affecting both left and right ventricles, with fibrosis that involved the endocardium and subendocardial tissues and covering the apex, papillary muscles and posterior ventricular wall, leading to atrioventricular valve dysfunction.^18^,^19^

The objective of our study was to document the current frequency of EMF in Sudan by identifying and selecting cases from patients attending our echocardiography clinics in fourdifferent hospitals, and creating an EMF registry. However during the process of acquisition and analysis of images, it emerged that there were new echocardiographic features that had not been reported before. These findings are presented and discussed below.

## Methods

This study is a prospective, descriptive study, which started in January 2007 and is on-going. Patients attending our echocardiography sessions at Amal National Hospital from 2007 to 2009, and the Academy Teaching and Yastabshiroun Hospitals from 2010 to 2011 were included; however the majority of the selected patients were enrolled from the Heart Clinic in Khartoum, Sudan. Permission for the study was obtained from the ethics committees in the four centres and informed verbal consent was obtained from each patient.

Transthoracic echocardiography using Mylab30 (Esaote, Italy) was performed by SIK on all selected patients, using the American Society of Echocardiography (ASE) standards.^20^ Additional information was obtained from the Echo Manual by Oh, Seward and Tajik.^21^

The following standard echocardiographic views were used: parasternal long-axis (PLAX), short-axis (SAX), M-mode, apical two-, four- and five-chamber views (AP2, AP4, AP5) and the apical long-axis view (APLX). Additionally, a modified APLX view, obtained by angulating the probe medially and rotating counter-clockwise to focus on the recess between the posterior papillary muscle and the posterior mitral valve leaflet was used.

The diagnosis of EMF was made on cases that fulfilled the following echocardiographic features and definitions:

Generalised endomyocardial thickening and fibrosis of the apex, ventricular walls and papillary muscles, and atrioventricular (AV) valve incompetence.
Obliteration of the ventricular cavity by fibrous tissue, defined as left ventricular (LV) diastolic volume less than 40 ml, measured by the modified Simpson’s rule in AP4 view and right ventricular (RV) size less than 20 mm, measured by the mid-RV diameter in the AP4 view.
Left atrial (LA) volume was measured with the biplane method of disks (modified Simpson’s rule) from AP4 and AP2 views at ventricular end-diastole. Huge left atrium was defined as atrial volume of more than 70 ml.
Right atrium (RA) was quantified from the apical four-chamber view. The minor-axis diameter was measured according to ASE recommendations.^19^ Right atrial dilatation was assumed when the mid-axis diameter was more than 5.0 cm.

Diagnosis was made from a detailed initial examination and ascertained by two review examinations, carried out during the first four weeks after the case was identified.

Additional information was derived from targeted history, clinical examination, 12-lead electrocardiogram (ECG), chest X-ray and complete blood count, including total blood count, differential white cell count and absolute eosinophilia (> 600 cells/μl).

## Results

Out of 4 332 cases studied, 23 (0.5%) were found to have features of EMF. Of these 23 cases identified, two were from Amal Hospital, two from Yastabshiroun Hospital, five from Academy Teaching Hospital and 14 cases were from the Heart Clinic in Khartoum. Females constituted 52% and the age range was 24 to 67 years. All patients presented with dyspnoea grades III–IV, and advanced heart failure with gross fluid overload was seen in 54% of cases and ascites was seen in 30%.

ECG findings were non-specific; sinus tachycardia was found in 22% of patients, atrial abnormality in 43%, first-degree heart block in 39% and atrial fibrillation in 13% of patients. Chest X-ray findings were also non-specific and showed cardiomegaly in 92% of patients.

Haematological findings included absolute eosinophilia in three patients and five cases had iron deficiency. EMF was biventricular in 53%, left ventricular in 29% and right ventricular in 18% of cases. Echocardiographic features of EMF are summarised in Table 1.

**Table 1:** Frequencies of echocardiographic features of EMF (n = 23)

*Echocardiographic features*	*Percentage of patients*
Apical fibrosis	100
Ventricular wall fibrosis	100
Atrial enlargement	100
Atrioventricular valve regurgitation	100
Ventricular cavity obliteration	91
Pericardial effusion	87
Endocardium fibrous shelf formation	82
Restrictive flow pattern	76
Ascites	30
Thrombus formation	6

**Fig. 1.**
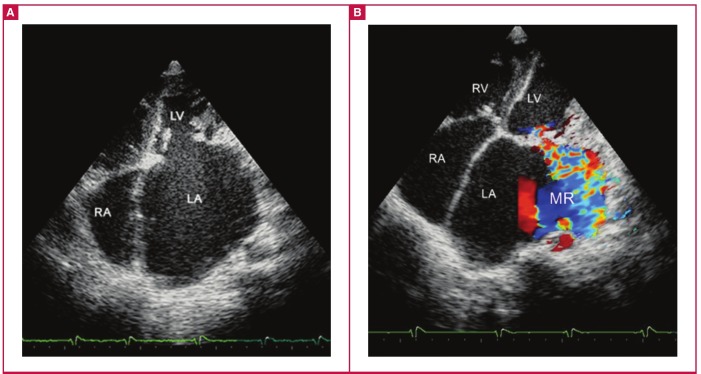
Basic echocardiographic features of left ventricular EMF. A and B, apical four-chamber view. The huge left atrium with apical and LV wall fibrosis, obliterated LV and mitral regurgitation (MR) (B), are the characteristic features of left ventricular EMF.

**Fig. 2.**
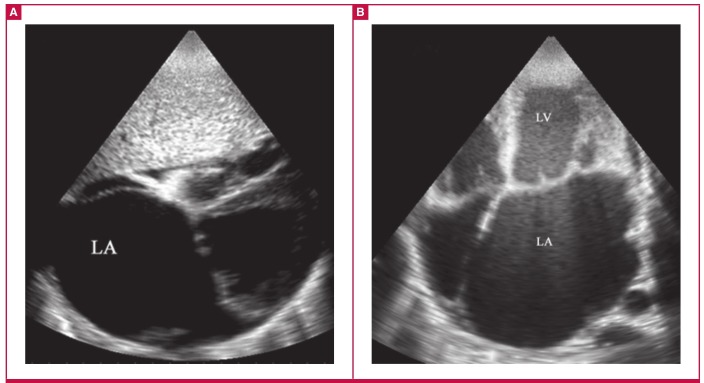
Basic echocardiographic features of advanced left ventricular EMF. Images from a 42-year-old female who presented with intractable heart failure. A is a subcostal view showing the typically huge left atrium occupying half the cardiac size. B is an AP four-chamber view showing apical fibrosis extending to the septum and ventricular walls, leading to severe obliteration of the left ventricular cavity.

**Fig. 3.**
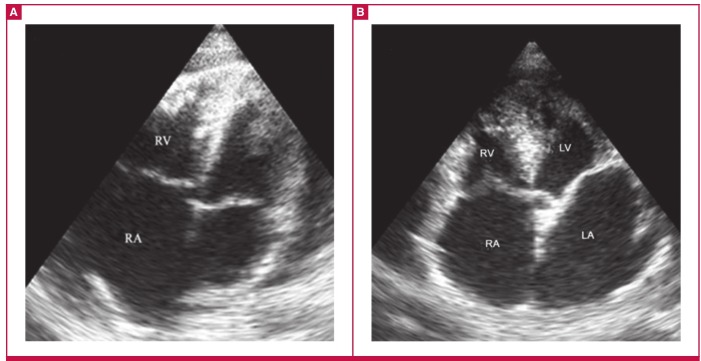
Right ventricular (RV) and biventricular EMF. A (AP4) showing EMF of the RV; note the apical fibrosis engulfing the moderator band, fibrosis of the anterior interventricular septum, dilated RA and obliterated RV. B (AP5) shows biventricular EMF

**Fig. 4.**
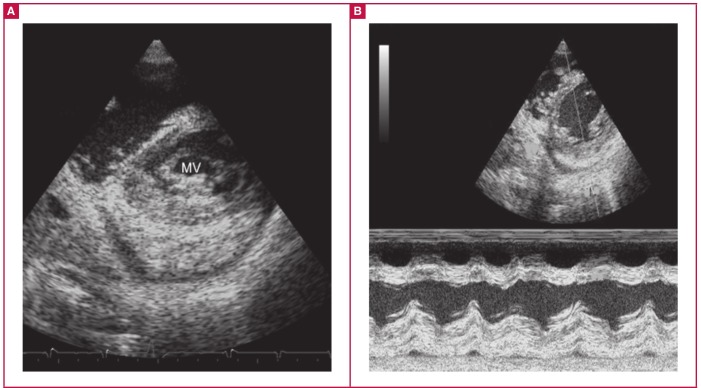
Layering. A is a short-axis view of a patient with advanced EMF showing layering of the thickened endocardium with the myocardium and pericardium. The posterior mitral valve leaflet (MV) is seen tethered to the endocardium. B is M-mode showing the distinct layers of the posterior wall with thickened endocardium and myocardium. A thickened pericardium with effusions can be seen peripherally.

**Fig. 5.**
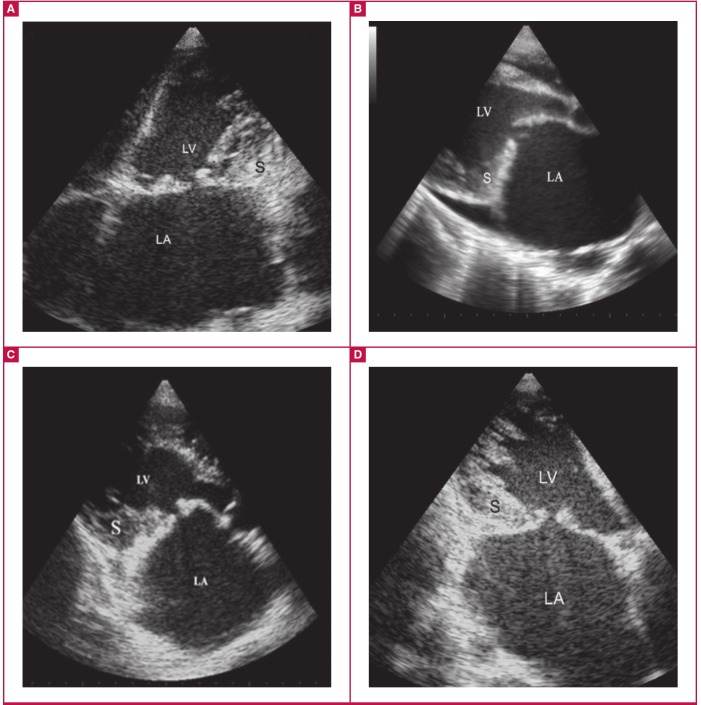
Endocardial fibrous shelf. A is PLAX, B is AP4, and C and D are modified APLX views from three different patients (B and D from same patient), with left ventricular EMF showing thickened endocardium spreading over the recess between the posterior papillary muscle and the posterior mitral valve leaflet, engulfing the leaflet and forming an immobile endocardial fibrous shelf (S). The anterior mitral valve leaflet although moderately thickened, moves freely, while the whole mitral structure appears reduced to a single leaflet valve.

**Fig. 6.**
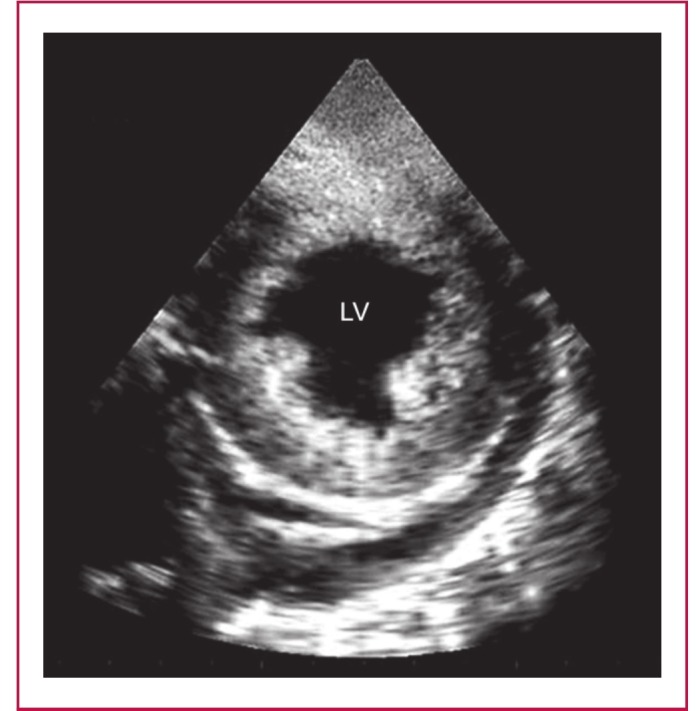
Endomyocardiopericardial fibrosis (EMPF). A shortaxis view from a patient with advanced EMF and intractable heart failure. The endocardium looks dense and bright with the myocardium clearly seen underneath it. The striking finding is the appearance of densely fibrosed and calcified pericardium with effusion forming an endomyocardiopericarial fibrosis. Note the presence of pericardial effusion and the posterior papillary muscle being engulfed by the thickened endocardium.

Echocardiographic images of EMF were divided into the following subgroups: images of basic echocardiographic features (covered in Figs 1–3), images of additional echocardiographic details (shown in Fig. 4) and images of new echocardiographic features (shown and described in Figs 5 and 6).

## Discussion

This descriptive study that discusses the clinical and echocardiographic features of EMF in Sudan is not intended to redefine the pathognomonic echocardiographic features of the disease, which have been well described in previous works.^3^,^4^,^6^,^22^ The main objective was to determine the current frequency of EMF in Sudan, as the last report on the disease dates back more than 40 years.

The method we used was to identify and select cases of EMF from patients attending our echocardiography laboratory using predefined features and definitions. All images relate to advanced forms of EMF, which were available in the hospital setting. We did not see mild or early forms of the disease and speculate that population-based studies are more appropriate for reporting these types of EMF.^4^

As we recognised only 23 cases of EMF during the course of eight years, we infer that the disease is rare in Sudan and that only isolated cases are prevalent. Consequently, a study of this nature will help improve the awareness of physicians to diagnose this disease.

The basic diagnostic echocardiographic features are shown in Figs 1 to 3. The images of apical and ventricular wall fibrosis together with huge atria should alert the investigator to the possibility of EMF. The presence of moderate-to-severe atrioventricular valve regurgitation and obliterated ventricles should provide further confirmation of the diagnosis (Fig. 1A, Fig. 2B, C).

These findings conform to features described in previous works.^4^,^6^,^23^ Right ventricular EMF echocardiographic features included fibrosis of the apex, right ventricular free wall and anterior interventricular septum with obliteration of the ventricular cavity and dilated right atrium (Fig. 3A). The pericardium was also found to be affected and looked fibrosed and thickened, with mild-to-moderate effusion in the majority of cases (87%). Severe effusion (32 mm) was noted in one case, without evidence of cardiac tamponade, possibly due to the chronicity of the disease.

As a result of fibrosis, the three layers forming the heart wall were identified as separate layers, especially in the posterior ventricular wall. This feature resulted in a form of ‘layering’ and is shown in Fig. 3 in both the short-axis (3A) and M-mode views (3B). Layering can be seen on all left ventricular walls, specifically the lateral and posterior walls. Although the thick fibrosis of the three separate layers was recognised in post mortem findings reported by Davies,^24^ echocardiographic images of layering have not been reported before.

## New echocardiographic features

Endocardial fibrous shelf: in countries where EMF is prevalent, the rates of rheumatic heart disease are also high and diagnostic difficulties arise in differentiating patients with mitral stenosis from those with EMF. A new echocardiographic feature, the endocardial fibrous shelf (EFS) seen in Fig. 5A–D provides useful diagnostic help.

These echocardiographic images correlate well with a previously recognised pathological finding first described by Davies in 1955. He reported that the posterior mitral cusp was completely immobilised by adherence to the endocardium of the posterior wall of the ventricle, and the end result was a fibrous surface running straight down from the atrium to the ventricle where the cusp had become embedded.^22^ Davies further added that in other cases, the remains of the cusp projected as a short, thick shelf. This finding is shown echocardiographically by an immobile posterior mitral leaflet tethered to the endocardium and appearing like a solid shelf. The anterior mitral valve leaflet, although moderately thick, moves freely, while the whole mitral structure becomes reduced to a single leaflet valve.

The echocardiographic endocardial fibrous shelf can be visualised in the modified APLX in all cases of left ventricular and biventricular EMF and provides a mark for differentiation between EMF of the left ventricle and rheumatic mitral stenosis, where the leaflets and subvalvular structure are fibrosed and may be calcified but the posterior LV wall remains free.

Endomyocardiopericardial fibrosis: in three cases of advanced EMF, a dense fibrous pericardium and pericardial calcification were seen (Fig. 6). This entity behaved clinically like constrictive pericarditis, as the three patients presented with tachycardia, ascites and gross oedema of the ankles. We opted to give this type of EMF, in which the pericardium played a significant pathological and clinical role, the name endomyocardiopericardial fibrosis (EMPF), and considered it a cause of pericardial constriction.

Although EMF is among the common causes of restrictive cardiomyopathy, its role in pericardial constriction has not been described before. Despite the fact that an endomyocardial biopsy from patients with both tuberculous constrictive pericarditis and endomyocardial fibrosis revealed similar histopathological changes of endocardial thickening and focal myofibrosis, evidence to support pericardial constriction in EMF could not be confirmed.^25^ The echocardiographic and clinical presentation of patients with EMPF lends support to pericardial constriction in association with EMF.

The differentiation of EMF from hypertrophic cardiomyopathy (HCM), especially the apical type, can be difficult. However our observations are consistent with the view of Fawzy, Ziady and Halilm in that with EMF, apical obliteration appears during both systole and diastole, in contrast to HCM where it occurs only in systole.^26^

One additional observation is the characteristically huge left atrium (91 mm in one case), which could not be seen, even in cases of severe mitral stenosis. Among the explanations offered were the obliteration of the ventricular cavity, and hence the increase in filling pressure, together with the additional volume load due to mitral regurgitation.

This study has provided high-definition images of the main diagnostic features of EMF. Images of layering provide additional identification of this multi-layer disease. This study has described and shown images of a new echocardiographic feature: the endocardial fibrous shelf, which offers an additional feature for left ventricular EMF. We also report a new entity, EMPF, a form of advanced EMF that clinically behaved like constrictive pericarditis.

One limitation of the study is that the new echocardiographic features reported in this study, namely endocardial fibrous shelf and endomyocardiopericarial fibrosis, were collected from a largely descriptive and small study, This needs further verification, which can only be achieved in a large study.

## Conclusion

This study confirmed that isolated cases of EMF are still seen in Sudan. The main features of EMF were apical and ventricular wall fibrosis, atrial enlargement and obliterated ventricles. New details on endomyocardial pericardial layering and images of endocardial fibrous shelf are described. A new echocardiographic entity, EMPF, an advanced form of EMF that behaves like constrictive pericarditis, was identified. Diagnosis of EMF needed a high index of suspicion to differentiate it from other conditions discussed in this article.
